# Concerns with Male Infertility Induced by Exposure to Titanium Nanoparticles and the Supporting Impact of *Pelargonium graveolens* Essential Oil: Morphometric Records in Male-Wistar Rats

**DOI:** 10.3390/life12050639

**Published:** 2022-04-26

**Authors:** Ahmed Abdou Said, Yasmin Nasr, Azza A. A. Galal, Ahmed E. Abdelhamid, Haiam A. Mohamed, Mohamed M. M. Metwally, Mahmoud A. Said, Mohamed A. Nassan, Naief Dahran, Amany Abdel-Rahman Mohamed

**Affiliations:** 1 Department of Pharmacology, Faculty of Veterinary Medicine, Zagazig University, Zagazig 44511, Egypt; saaid52@zu.edu.eg (A.A.S.); yasminn@zu.edu.eg (Y.N.); azzagalal@zu.edu.eg (A.A.A.G.); 2 Polymers & Pigments Department, National Research Centre, 33 EL Buhouth St., Dokki, Giza 12622, Egypt; ae.abdel-hamid@nrc.sci.eg; 3 Department of Physiology, Faculty of Veterinary Medicine, Zagazig University, Zagazig 44511, Egypt; haiamm@zu.edu.eg; 4 Department of Pathology, Faculty of Veterinary Medicine, Zagazig University, Zagazig 44511, Egypt; mmetwally@zu.edu.eg; 5 Medical Finance Department, Zagazig University Hospital, Zagazig 44519, Egypt; ph.mahmoud.91@zu.edu.eg; 6 Department of Clinical Laboratory Sciences, Turabah University College, Taif University, P.O. Box 11099, Taif 21944, Saudi Arabia; m.nassan@tu.edu.sa; 7 Department of Anatomy, Faculty of Medicine, University of Jeddah, Jeddah 21959, Saudi Arabia; ndahran@uj.edu.sa; 8 Department of Forensic Medicine and Toxicology, Zagazig University, Zagazig 44511, Egypt

**Keywords:** titanium nanoparticles, rose-scented geranium, steroidogenesis, testicular tissue, sex hormones, CYP17A1, TEM, DLS, oxidative stress, morphometry

## Abstract

*Background*: Due to the increased use of titanium dioxide nanoparticles (TiO_2_ NPs), the risks of their reprotoxic effect arise. This study anticipated examining the potential protective effects of GEO (geranium essential oil) components screened via GC/MS analysis against the reprotoxic impacts of TiO_2_ NPs on male rats. *Methods*: Thirty-two adult male rats were randomly assigned to four groups: control, GEO (75 mg/kg bwt/orally/day/60 days), TiO_2_ NPs (100 ppm/rat/IP/day/60 days), and TiO_2_ NPs + GEO. After 60 days, hormonal assay, semen appraisal, lipid peroxidation, antioxidant enzymes, testis and prostate morphometry, and the steroidogenesis-related genes’ mRNA expressions were assessed. *Results*: The TEM and DLS results demonstrated that synthesized TiO_2_ NPs are spherical with minimal aggregations polydispersed and varying in size from 50 to 100 nm. TiO_2_ NPs IP injection-induced sperm abnormalities decreased the percent of motile sperms in the sperm count, reduced sex hormone levels, altered the testicular oxidant/antioxidant status and mRNA expression of steroid-related genes, and induced architectural alterations in testicular, epididymal, and prostate gland tissues. GEO significantly rescued the TiO_2_ NPs-altered spermiogram, sex hormones, and antioxidant capacity, restored the tissue architectures, and enhanced steroidogenesis-related gene mRNA expression. *Conclusions*: These findings may significantly contribute to developing combinatorial treatments for infertility associated with various environmental and industrial xenobiotic exposures.

## 1. Introduction

With the advancement of nanotechnology, there has been a remarkable increase in the use of nanoparticle applications (NPs) for drug delivery systems, industries, and cosmetics. Titanium dioxide nanoparticles (TiO_2_ NPs) are manufactured globally in significant quantities for worldwide use in various applications, including home requirements, paints, plastics, rubber, pharmaceutical products, food colorants, and white pigment [1,2]. In addition, TiO_2_ NPs are widely used in printing ink, sunscreens, paper, car materials, and water purification [3,4]. The difference in size between the TiO_2_ particles and TiO_2_ NPs has been utilized in different eras. Due to these same features, there have been many health concerns about TiO_2_ NPs’ unique bioactivity. [5]. Several studies have demonstrated that TiO_2_ NPs are more toxic than fine and conventional titanium particles [6]. The toxicity of TiO_2_ NPs has been attributed primarily to the formation and accumulation of reactive oxygen species (ROS), which enhances an inflammatory response [7], exhaustion of cellular antioxidants, for instance, glutathione [8], and mitochondrial damage with the impediment of ATP synthesis [9].

Male fertility is typically determined by the amount and quality of spermatozoa, adequate activity of Leydig cells, and a sufficient hormonal balance [10]. Infertility is a prevalent condition characterized by the inability to conceive after one year of unprotected intercourse, with no visible signs of infertility [11]. The disruption of critical cellular mechanisms required for proper reproductive activities contributes significantly to the problem [12]. Enormous production and release of industrial chemicals into the environment has caused the scientific community to postulate that present contaminants may irrefutably disturb health conditions, leading to substantial harm to the physiological functioning of reproduction [13,14]. Some earlier reports revealed the degenerative effect of TiO_2_ NPs on testicular tissue, as evidenced by the altered expression of genes involved in steroidogenesis, glutathione-S-transferase (GST), interleukin-6 (IL-6), tumor necrosis factor-alpha (TNF-α) [15], and activated caspase-3 and -9 [16] after oral exposure. 

Rose-scented geranium (*Pelargonium graveolens*) is generally a medicinal plant with the most significant antioxidant activity [17]. Essential oils are a folk medicine, and recently, their usage has increased globally to include therapy against several forms of inflammatory disorders [18]. Geranium essential oil (GEO) has a high capacity to trap free radicals and restrict the production of new free radicals, lowering oxidative stress and inhibiting the development of several fatal consequences [17]. GEO has historically been used in the French medicinal community to treat several disorders, including liver problems, diarrhea, gastric ulcers, gallbladder problems, and sterility [19,20]. In Chinese homeopathy, GEO is known to open up the liver charka and stimulate the outflow of toxins that impair the internal balance [17]. 

This research will provide a better understanding of how natural oils can be used to combat oxidative stress and reprotoxicity caused by some known toxins like TiO_2_ nanoparticles that might impair reproductive organ functions, as well as the mechanisms through which these oils can fulfill their protective role. 

The purpose of the current study was to describe the components of *Pelargonium graveolens* essential oil and investigate their protective potential against TiO_2_ NPs-induced reprotoxicity in male Wistar rats. Therefore, semen, testicular antioxidant enzymes, hormonal levels, the mRNA expression of steroidogenesis, mitochondria and biogenesis-related genes were assessed. In addition, a histological evaluation of testicular and epididymal tissues and a prostate gland and morphometric appraisal was done. 

## 2. Materials and Methods

### 2.1. Synthesis of TiO_2_ Nanoparticles 

TiO_2_ nanoparticles were synthesized using the hydrothermal method, as previously reported in the literature [21]. Briefly, 0.1 N of titanium tetra-isopropoxide (Merck) in ethanol (20 mL) was stirred for 30 min to form the dispersion medium via a few drops of distilled water. The obtained dispersion was sonicated for approximately 20 min in an ultrasonic bath before being placed in an autoclave at 150 °C for 3 h. After allowing the solution to cool down to room temperature and removing the impurities, it was centrifuged, washed with deionized water, and filtered with filter paper (Whatman No. 1). The filtered material was dried in an oven at 110 °C for about 5 h, then annealed at 500 °C for approximately 2 h. The TiO_2_ NPs produced were characterized using a transmission electron microscope (TEM) and dynamic light scattering (DLS).

### 2.2. Transmission Electron Microscope (TEM)

The Zeta Sizer instrument can determine particle size and Zeta potential (HR-TEM, JEOL-JEM-2100). After sonicating the dilute nanoparticles suspension for an hour, one or two drops were dropped onto the testing grid and left for drying [22,23].

### 2.3. Dynamic Light Scattering (DLS)

The particle size and Zeta potential can be measured using the Zeta Sizer instrument (Nano-ZS, Malvern Instruments Ltd., Zetasizer Ver, 704, Worcestershire, UK). The dilute suspension of the prepared nanoparticles was firstly sonicated to ensure good dispersion of nanoparticles in aqueous media and then measured directly using DLS based on the scattered beam resulting from the Brownian motion of dispersed particles in a solution [24]. 

### 2.4. Plant Essential Oil Extraction and Gas Chromatography/Mass Spectrometry Analysis (GC-MS)

The leaves of the Pelargonium graveolens were obtained from the field station of the Medicinal and Aromatic Plants Research Department, which is located at the Horticulture Research Institute, El-Kanater El-Kharia city, Qalyubia Governorate, Egypt. The oil was extracted according to the procedure given by [25]. The GC-MS analysis of GEO was conducted as described in the previous work in which the analysis of GEO oil is detailed by [26]. The GEO was stored in a dark glass container at 4 °C until GC–MS analysis and biological activity testing were utilized.

### 2.5. Experimental Design

**Figure 1:**
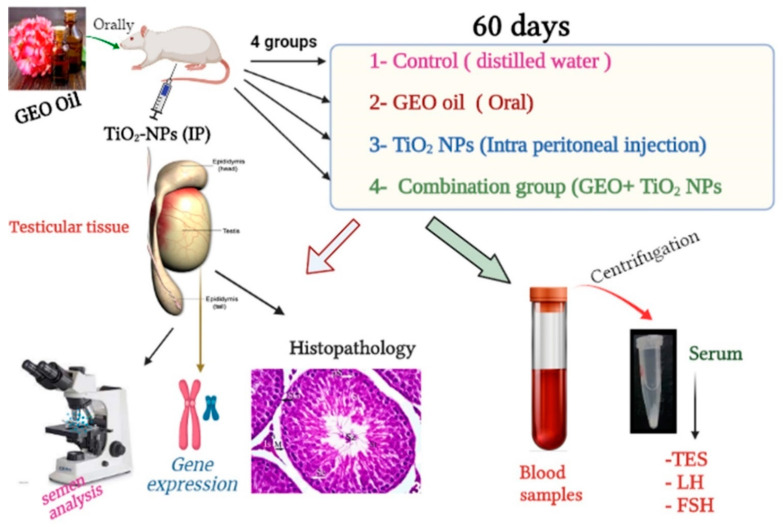
A diagram describing the experimental design (groups, treatments, duration, and laboratory investigations).

The current study utilized thirty two adult male Wistar albino rats (Rattus norvegicus) weighing 150–160 g procured from Zagazig University’s Faculty of Veterinary Medicine. They were housed in metal cages at a temperature of 23.2 °C with a 40–60% humidity range and a 12 h light/dark cycle. They were fed a rodent diet and given unlimited access to water throughout the experiment. The rats were allowed to adjust to their new surroundings for a total of two weeks. The Guide for the Care and Use of Laboratory Animals National Institutes of Health guidelines in Zagazig University, Egypt IACUC committee, under the reference number (ZU-IACUC/2/F/611/2021) was followed to the letter in the housing and care of the animals and in the design of the experiments. After the accommodation period, rats were weighed and randomly allocated into four equal groups. Group I (C): Control group, received distilled water (DW). Group II (GEO): received geranium essential oil (GEO) orally at a dose of 75 mg/kg BW [27]. Group III (TiO_2_ NPs): intraperitoneally injected )IP) with TiO_2_ NPs at a dose of 100 ppm/rat [28]. Group IV (GEO + TiO_2_ NPs): pre-treated with GEO then TiO_2_ NPs, 1 h later. The experiment lasted for 60 days for all treated groups and controls. Figure 1 summarizes the different groups and different laboratory investigations. 

### 2.6. Dose Selection Strategy

In the present study, the TiO_2_ NPs dose was selected since it induced moderate to severe damage to numerous organs when evaluated even for a short period (7–14 days) in a previously published study, similar to what occurred in the liver [29]. A lower dose (60 parts per million of titanium dioxide nanofiber [TDNF] for 2 weeks) was previously tested on the kidney [29]. The exposure occurring for industrial workers usually lasts too long. Therefore, it is strongly advised to apply numerous studies for longer durations to cope with what is occurring in the same environmental circumstances and conclude the risks specifically on male reproductive organs. The dose selection of GEO oil was decided based on the earlier work of [29], where the GEO administration performed correctly as an efficient antioxidant candidate to reduce the oxidative stress problems produced by alloxan-induced diabetic Mellitus in a rat model. As a result, we hypothesized to estimate the same dose to overcome the oxidative damage induced by TiO_2_ NPS in the testicular tissue of rats.

For a total of 60 days, the groups received various treatments. Throughout the experiment, rats were closely monitored for signs of toxicity and mortality. At the end of the experiment, rats were weighed and anesthetized with a 50 mg/kg ketamine hydrochloride/xylazine mixture intramuscularly. In this study, blood was drawn from the retro-orbital venous plexus of rats and centrifuged in tubes that had been cleaned, autoclaved, dried, and labelled. In order to obtain the serum, blood was allowed to coagulate for 15 min before being centrifuged at 3000 rpm. The clear serum was stored at −20 °C until used for hormonal and biochemical analysis. After blood sample collection, the rats were sacrificed, and the testis, epididymis, and prostate were immediately removed. The right testes were prepared for light microscopy, and the remaining testes were kept at −80 °C until the preparation of tissue homogenates and gene expression analysis. 

### 2.7. Sperm Motility Assay, Count, and Morphological Abnormalities

Cauda epididymis sperm from one rat testis was extracted and placed in a sanitized Petri plate containing 2 millilitres of warm (37 °C) normal saline for further testing and analysis. A small hole in the epididymal content was cut with sterilized scissors [11]. One drop of the sperm suspension was placed on a glass slide to canvas 200 motile sperm in four different fields [30]. 

Sperm motility: The epididymal suspension was extracted from the cauda epididymis of the rat’s testis by dissolving the epididymis in 2 mL of normal saline in a sterile Petri plate (37 °C) [11]. This solution was evaluated microscopically for the percentage of sperm motility by performing an analysis of 200 sperms in different microscopic fields within 2–4 min after their extraction from the epididymis, and the data are expressed as percentages [31]. 

Sperm concentration: In order to kill the spermatozoa, a few drops of 40 percent formalin were added to the sperm suspension, which was then carefully mixed before being used to count the sperm with a hemacytometer counting chamber (Thoma chamber, 0.0025 mm^2^ and 0.100 mm depth, Germany). Counting (spermatozoa/mL) was carried out as previously reported. [30].

The percentage of sperm abnormalities has been assessed as previously described in previously published articles [32]. Briefly, the formalin-treated semen solution was combined with two drops of eosin–nigrosine stain on a microscope slide, smeared, dried in the air, and analyzed. Two hundred spermatozoa were randomly selected and analyzed for each sample for various anomalies. 

### 2.8. Hormonal Analysis

A rat-specific ELISA kit from MyBioSource (San Diego, CA, USA) was used to detect TES, FSH, and LH in the serum of rats in each of the study groups. The kit was specifically designed for use with rat sera. The competitive-ELISA detection method of these hormones was used to develop these kits with a target-coated microtiter plate. The biotinylated detection antibody specific to targets competes with a set number of targets, which are the detected hormone (LH) on the solid phase supporter. HRP-streptavidin (SABC) was applied to each microplate well and incubated after excess conjugate and unbound samples or standards had been rinsed from the plate. Subsequently, each well was filled with a TMB substrate solution. The color change was detected spectrophotometrically at a wavelength of 450 nm after the addition of a sulphuric acid solution to the enzyme–substrate reaction. We managed to calculate the target concentration in each of our test samples by comparing the samples’ optical density (OD) to that of a standard curve. Kits had a sensitivity of (0.04 ng/mL) for TES, (0.938 mIU/mL) for LH, and 1.88 ng/mL for FSH. 

### 2.9. Testicular Oxidative/Antioxidant Status

Testes were homogenized in 5 mL cold phosphate buffer (pH 7.4). Glutathione peroxidase (GPx) was detected following the method of Beutler, et al. [33], superoxide dismutase (SOD), and catalase (CAT) according to [34] and Aebi [35], respectively. Malondialdehyde (MDA) was also detected following the protocol of Mihara and Uchiyama [36].

### 2.10. Gene Expression

Tissue samples were used to extract RNA for assessment of the expression of steroidogenesis-related genes in the rat model, including cytochrome P450 Family 17 Subfamily A (CYP17-A1), steroidogenic acute regulatory protein (STAR), 17-beta hydroxysteroid dehydrogenase 3 Member 1 (HSD 17-B3), and peroxisome proliferator-activated receptor-gamma coactivator 1-alpha (PGC1). One volume of 70% ethanol was added according to the Purification of Total RNA protocol of the QIAamp RNeasy Mini kit (Qiagen, Hilden, Germany, GmbH) to clarify the lysate. DNase digestion was performed on column DNase to remove residual DNA. The primers supplied by Metabion (Bayern, Germany) are listed in Table 1. Primers were utilized in a 25-µL reaction containing 10 µL of the 2X *HERA* SYBR**^®^** Green RT-qPCR Master Mix (Willowfort, Nottinghamshire, UK), 1 µL of RT Enzyme Mix (20X), 0.5 µL of each primer of 20 pmol concentration, 5 µL of water, and 3 µL of RNA template. The reaction was completed by a step one real-time PCR machine. In the first step, software generated amplification curves and ct values. The CT of each sample was compared to that of the positive control group to quantify the variation in gene expression on the RNA of the various samples utilizing the “ΔΔCt” method described by [37]. 

### 2.11. Histopathological Investigations 

Representative tissue specimens from the testes, epididymies, and prostates were sampled after death following the guidelines of [38] and fixed overnight in 10% neutral phosphate-buffered formalin. They were subsequently processed for the paraffin technique, sectioned at 5 μm, and stained with hematoxylin and eosin [39] for histological examination. Testicular morphometric analysis was conducted, including (1) the numbers of seminiferous tubules (ST) were counted in a 10× microscopic field; (2) the mean diameters of ST and the heights of the germinal epithelium were carried out in 10 seminiferous tubules per animal and were obtained by using the opensource ImageJ software version 1.41; and (3) the numbers of spermatogonia, spermatocytes, spermatids, and Sertoli cells/ST were calculated in 15 randomly selected seminiferous tubules (regardless the stage of spermatogenesis) per animal. A high-power field (40 objective) was used for the counting of the germ and Sertoli cells because the differentiation between the germinal epithelial cells was dependent upon cellular location and nuclear size, shape, location, and chromatin pattern. In addition, a multiparametric lesion scoring of testicular abnormalities (the frequencies of histopathological alterations/images and the proportion of ST showing basement membrane abnormalities, tubular atrophy, germ cell depletion, vacuolation, desquamation, necrosis, and multinucleated giant cells in relation to the total number of ST/images) was carried out in five nonoverlapped 10× microscopical fields. The epididymal lesion scoring different pathologies (tubular irregularity, reduced or absence of luminal sperms, presence of luminal round spermatids and/or exfoliated material, loss or disrupted of stereocilia, vacuole formation, necrosis, desquamation, hyperplastic alteration, interstitial inflammatory infiltrates and/or vascular congestions or hemorrhages) was carried out in five nonoverlapped 4× microscopical fields. The prostatic lesion scoring different pathologies (reduced or absence of luminal secretion, reduced acinar lumina, acinar dilatation, crowding or absence of the intra-acinar epithelial folds, vacuolar degeneration, necrosis or apoptosis of the epithelial lining, hyperplasia of acinar epithelium, and interstitial inflammatory cell infiltrate, fibrosis, congestion, edema, and hemorrhage) was carried out in five nonoverlapped 4× microscopical fields. All measurements were performed using AmScope Toup View v4.11.19627 software, AmScope, Irvine, CA, USA, and the ImageJ software version 1.41. The results were presented as percentages (means ± SE).

### 2.12. Data Analysis

All data were verified for normality by the Shapiro–Wilk test and Levene’s mean test for homogeneity and normality of variance identified before being analyzed. Data are expressed as the means ± the standard error (SE). Biochemical assays were compared statistically using one one-way ANOVA in SPSS 21.0. Duncan’s multiple comparison post hoc test was applied to compare the mean values of treated and control groups. *p* values < 0.05 were considered statistically significant (N = 6/group).

## 3. Results

### 3.1. Characterization of Nanoparticle of TiO_2_

**Figure 2:**
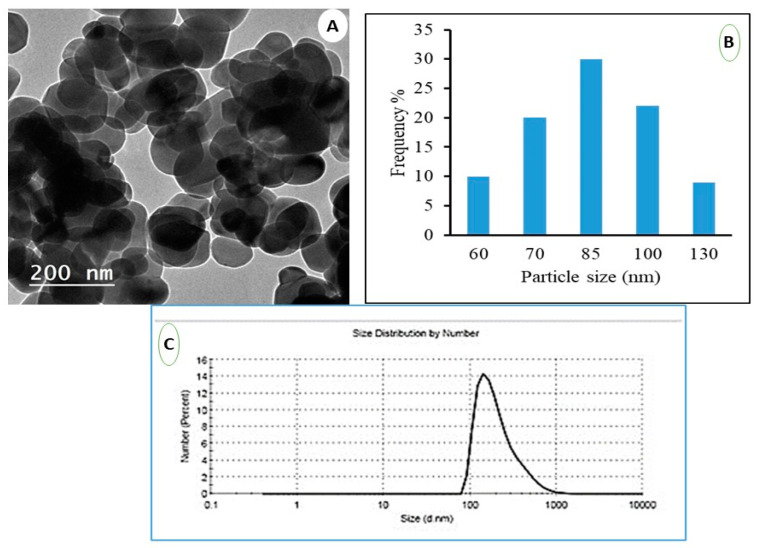
The characterization of the TiO_2_ NPs. (**A**) TEM of TiO_2_ nanoparticles, (**B**) corresponding histogram from image analysis and (**C**) DLS of the prepared TiO_2_ nanoparticles.

TiO_2_ nanoparticles were produced as indicated in Figure 2A. Nanoparticles generated using the hydrothermal method have a spherical shape with a small aggregation. They had an average particle size of between 50 and 100 nm, with an average of about 85 nm, as demonstrated in the histogram (Figure 2B) obtained from image analysis using ImageJ software. TiO_2_ nanoparticle synthesis using the hydrothermal approach can produce tunable nanoparticles of various shapes and excellent yields. Dynamic light scattering (DLS) of TiO_2_ nanoparticles was depicted in Figure 2C. The particle size was 140 nm, as depicted in the image. The particle sizes revealed by DLS measurements are significantly larger than those revealed by TEM measurements. Overall, DLS was used to determine the hydrodynamic radius of nanoparticles (hydrated particles) in an aqueous solution, whereas the dry diameter of nanoparticles was provided by TEM. The TEM and DLS, on the contrary, both provide images of the nanoparticles and the aggregates they form in a specific measurement area. In addition, the TEM provides an image of the selected area for measurement, while DLS provides an overall image of the nanoparticles and their aggregations. 

### 3.2. GC-MS Analysis of GEO Essential Oils 

Table 2 displays the results of the GC-MS analysis of GEO, including peak area percentages and retention periods. Citronellol (37.24%) and geraniol (12.32%) were the most prevalent chemicals found in GEO oil, followed by D-isomenthone, which mainly was reserved for fragrance, flavor, and pharma in usage applications (6.17%); citronellyl ester (9.35%); and epi-γ-Eudesmol (6.87). There were abundant components and various fractions of several components regarded as important antioxidants and flavonoids.

### 3.3. Effect of TiO_2_ NPs and/or GEO Essential Oil Administration on Spermiogram

**Figure 3:**
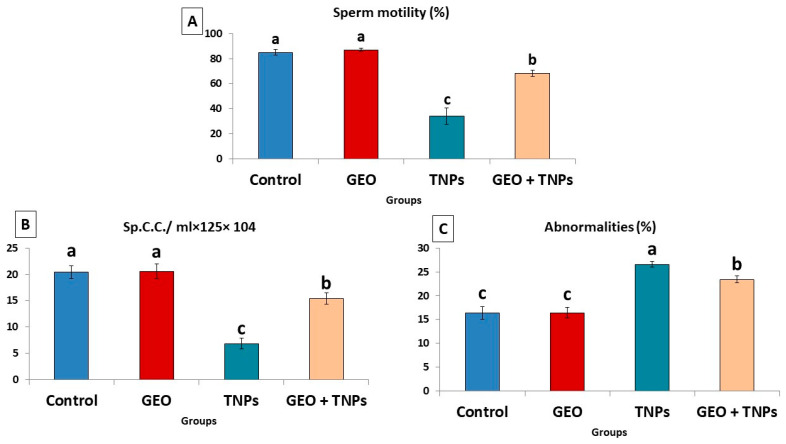
Effect of titanium dioxide nanoparticles (TiO_2_ NPs) delivered by intraperitoneal injection (100 ppm/rat) and/or geranium essential oil (GEO) delivered orally (75 mg/kg bwt/day, 60 days), on sperm characteristics, including (**A**) sperm motility, (**B**) sperm concentration and (**C**) total sperm abnormalities in male rats. Data expressed as mean ± SE, N = 8 for each group. Each bar carrying different letters (a, b, and c) was significantly different at *p* < 0.05.

As demonstrated in Figure 3, the TiO_2_ NPs-administered group displayed a significant decrease (*p* < 0.05) in sperm motility (34.00 ± 6.7) and sperm concentration (6.8 ± 1.02) compared to the control group. However, the percentage of sperm abnormalities increased significantly (*p* < 0.05) in the TiO_2_ NPs rats compared to the control group (Figure 3A). Many abnormalities included curved tail, looped tail, detached head, detached tail, broken head, hookless head, looped tail, as well as detached and curved tail. Nevertheless, GEO oral dosing combination treatment with TiO_2_ NPs substantially elevated the sperm count and motility while decreasing the proportion of sperm abnormalities compared to the TiO_2_ NPs-exposed group.

### 3.4. Effect of TiO_2_ NPs and GEO Essential Oil Administration on Male Reproductive Hormones

**Figure 4:**
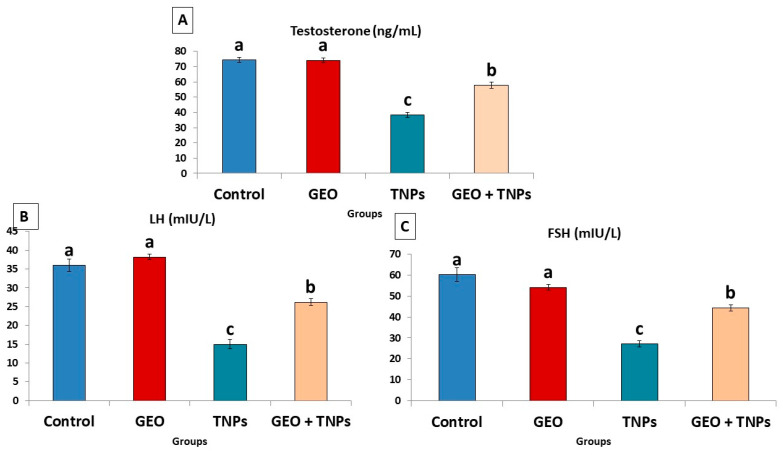
Effect of titanium dioxide nanoparticles (TiO_2_ NPs) delivered by intraperitoneal injection (100 ppm/rat) and/or Geranium essential oil (GEO) delivered orally (75 mg/kg bwt/day, 60 days), on sexual hormonal variables including (**A**) testosterone (TES), (**B**) follicle-stimulating hormone (FSH), and (**C**) luteinizing hormone (LH) levels in the serum of male rats. Data expressed as mean ± SE, N = 8 for each group. Each bar carrying different letters (a, b, and c) was significantly different at *p* < 0.05.

In the rats exposed to TiO_2_ NPs, FSH, TES, and LH, levels decreased by 54.8%, 48.24%, 58.33%, and 68.54%, respectively, compared to the control group (DW) (Figure 4). Nonetheless, treating rats with GEO oil concurrently with TiO_2_ NPs significantly (*p* < 0.05) improved the levels of the sexual hormone concentration compared to the TiO_2_ NPs-exposed group.

### 3.5. Effect of TiO_2_ NPs and GEO Essential Oil Administration on Testicular Lipid Peroxidation Level and Antioxidant Enzymes

**Figure 5:**
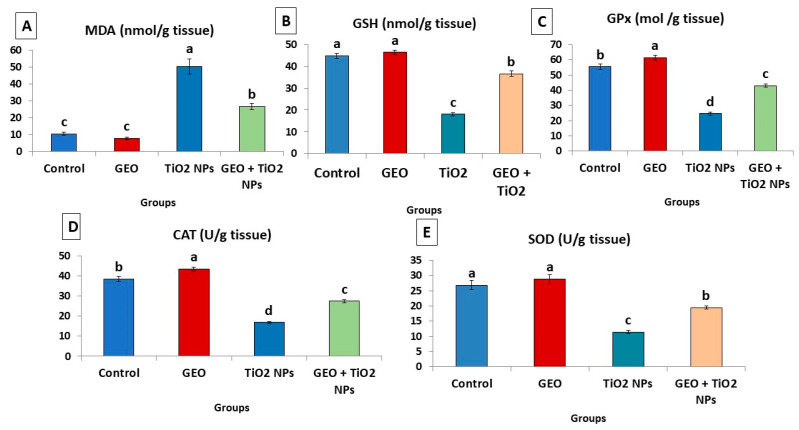
Effect of titanium dioxide nanoparticles (TiO_2_ NPs) delivered by intraperitoneal injection (100 ppm/rat) and/or geranium essential oil (GEO) delivered orally (75 mg/kg bwt/day, 60 days), on (**A**) malondialdehyde (MDA), (**B**) reduced glutathione (GSH), (**C**) glutathione peroxidase (GPX), (**D**) catalase (CAT), and (**E**) superoxide dismutase (SOD) levels in the testicular tissues of male rats. Data expressed as mean ± SE, N = 8 for each group. Each bar carrying different letters (a, b, c and d) was significantly different at *p* < 0.05.

As demonstrated in Figure 5, the testicular tissues of the TiO_2_ NPs group showed a significant (*p* < 0.05) increase in the detected level of MDA by about (3.7 folds) compared to the control group. Nevertheless, depletion of the enzymatic antioxidants (CAT, GPx, and SOD) by 56.25%, 55.59%, and 57.46%, respectively, and non-enzymatic antioxidants (GSH, 52.51%), was more evident in the TiO_2_ NPs-group than the control group. However, the presented data showed that the oral administration of GEO concurrently with TiO_2_ NPs significantly (*p* < 0.05) restored antioxidant levels in testicular tissue by 41.23% in SOD, 42.79% GPx, 38.68% CAT, and 103% in GSH and decreased the concentration of MDA by 46.82% compared to the TiO_2_ NPs group.

### 3.6. Effects of TiO_2_ NPs and/or GEO Essential Oil Administration on the Testicular Gene Expression 

**Figure 6:**
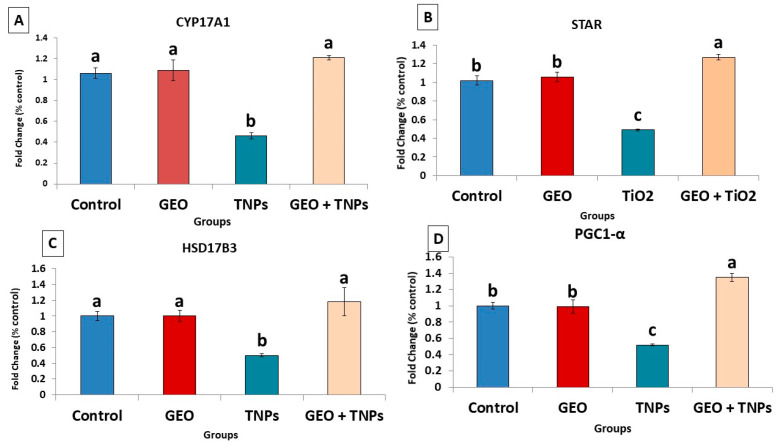
Effect of titanium dioxide nanoparticles (TiO_2_ NPs) delivered by intraperitoneal injection (100 ppm/rat) and/or geranium essential oil (GEO) delivered orally (75 mg/kg bwt/day, 60 days) on mRNA expression of (**A**) cytochrome17A1 (CYP17A1), (**B**) steroidogenic acute regulatory protein (StAr), (**C**) hydroxysteroid 17-beta dehydrogenase 3 (HSD17B3), and (**D**) peroxisome proliferator-activated receptor gamma coactivator 1-alpha (PGC-1α) in the testicular tissues of male rats. Data expressed as mean ± SE, N = 8 for each group. Each bar carrying different letters (a, b, and c) was significantly different at *p* < 0.05.

TiO_2_ NPs exposure on the steroidogenesis-related genes in testicular tissue showed significant (*p* < 0.05) downregulation (0.50 ± 0.02, 0.49 ± 0.01, and 0.46 ± 0.03,) in 17ΒHSD, StAr, and CYP17A1, compared to the control group (1.00 ± 0.08, 1.00 ± 0.10, and 1.00 ± 0.09, respectively) (Figure 6). Furthermore, when compared to the control group (1.00 ±0.07), the mitochondrial biogenesis-related gene PGC was significantly (*p* < 0.05) downregulated (0.52 ± 0.01) due to TiO_2_ NPs-exposure. When GEO was administered concurrently with TiO_2_ NPs, the mRNA expression pattern of the investigated mitochondrial biogenesis and steroidogenesis-related genes improved significantly (*p* < 0.05) compared to the TiO_2_ NPs-exposed group.

### 3.7. Histopathological Findings 

#### 3.7.1. Testes

**Figure 7:**
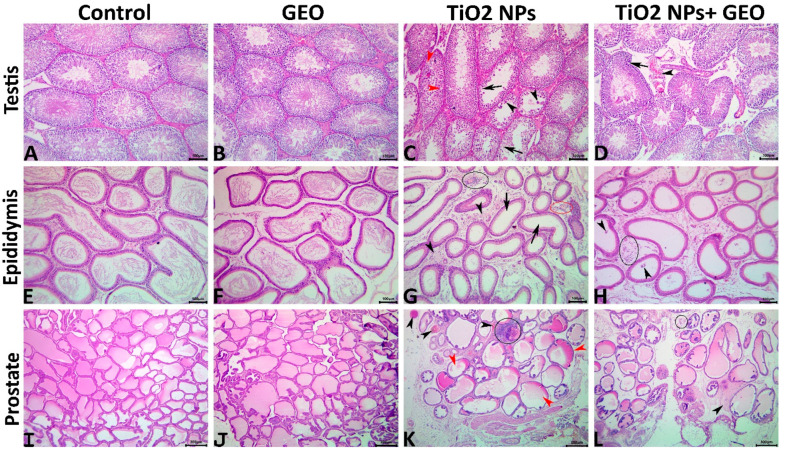
(**A**–**D**) Representative photomicrographs of the hematoxylin- and eosin-stained testicular tissue sections showing normal histological pictures in control (**A**), and GEO-treated (**B**) rats. The TiO_2_ NPs-treated rats showing vacuolated (red arrowheads), depleted and necrotic (black arrows) seminiferous epithelium, and multinucleated giant cells (black arrowheads) (**C**). The GEO TiO_2_ + NPs-treated rats showing irregular basement membrane (arrow) and interstitial congestion (arrowhead) (**D**). (**E**–**H**) Representative photomicrographs of the hematoxylin- and eosin-stained epididymal tissue sections showing normal histological pictures in control (**E**) and GEO-treated (**F**) rats. The TiO_2_ NPs-treated rats showing absence of luminal sperm cells (arrows), interstitial inflammatory cell infiltrate (black ellipse), vascular congestion (red ellipse), and increased connective tissue elements (arrowheads) (**G**). The GEO TiO_2_ + NPs-treated rats, showing the presence of round spermatids (arrowheads) and interstitial few inflammatory cells infiltrate (arrow) (**H**). (**I**–**L**); Representative photomicrographs of the hematoxylin- and eosin-stained prostatic tissue sections showing normal histological pictures in control (**I**), and GEO-treated (**J**) rats. The TiO_2_ NPs-treated rats showing numerous acini with inhomogeneous contents (red arrowheads), vascular congestions (black arrowheads), and focal inflammatory cell aggregate (ellipse) (**K**). The GEO TiO_2_ + NPs -treated rats showing interstitial edema (arrowhead), and minute inflammatory cell infiltrate (arrow head) (**L**).

In the control and GEO-treated rats, the testes had well-organized STs with successive populations of maturing germinal epithelial cells (spermatogonia, spermatocytes, round spermatids, and elongating spermatids) interspersed with basally located Sertoli cells and surrounded by intertubular loose connective tissue (Figure 7A,B). In the TiO_2_ NPs group, mild to moderate degenerative alterations in the seminiferous epithelium besides mild circulatory changes in the intertubular interstitium were evident. Some STs demonstrated impaired integrity, such as irregular basement membrane, the presence of luminal multinucleated giant cells, vacuolated, necrotic, and detached epithelium, along with increased numbers of STs/microscopic felid, and significant decreases in their mean diameters, epithelium heights, numbers of spermatogonia, spermatocytes, spermatids, and Sertoli cells/ST (Figure 7C). No inflammatory cell infiltrates, but vascular congestions and edema with increased connective tissue elements were detectable in some tissue sections’ intertubular interstitium. Supplementation with GEO did not maintain the normal tubular integrity; however, a significant reduction in the occurrence and degree of the TiO_2_ NPs-induced morphological changes was evident in the GEO + TiO_2_ NPs-treated rats. The most prominent lesions in this group were basement membrane irregularities, vacuolation, seminiferous epithelial degeneration, interstitial vascular congestions, and expanded intertubular areas (Table 3). The testicular morphometric analysis and lesion scoring among all groups are summarized in Table 3.

#### 3.7.2. Epididymis 

The epididymal ducts of the control and GEO-treated rats displayed normal histological architectures (Figure 7E,F). The epididymies of the TiO_2_ NPs-treated animals manifested numerous degenerative and inflammatory alterations, including tubular irregularity, presence of luminal round spermatids, decrease and absence of the luminal sperms, decrease in the tubular diameters, loss of stereocilia, vacuole formation, interstitial inflammatory infiltrates, edema and vascular congestions, besides an increase in the connective tissue elements (Figure 7G). The effects of GEO supplementation on the TiO_2_ NPs-induced epididymal histological alterations were evident, with significantly decreased integrative alterations, and the interstitial inflammatory or circulatory alterations were recorded in the GEO + TiO_2_ NPs-treated rats. Most tubules regained their normal diameters, but luminal sperm cells were reduced or absent, with the presence of round spermatids, minute interstitial inflammatory cell infiltrates, and edema still observable (Figure 7H). The epididymal lesion scoring among all groups is summarized in Table 3.

#### 3.7.3. Prostate 

The prostates of the control and GEO-treated rats had normal histological architectures (Figure 7I,J), whereas the prostates of the TiO_2_ NPs-treated animals demonstrated various morphological changes, including the acinar lining epithelium, acinar luminal contents, and interstitial tissue. Some acini showed reduced or absent luminal secretion with barely visible intra-acinar epithelial folds, and others were dilated with inhomogeneous eosinophilic luminal contents. The inter acinar interstitium showed notable inflammatory cell infiltrate, edema, minute hemorrhages, and vascular congestion (Figure 7K). The protective effects of GEO supplementation on prostate histology were apparent as a significant reduction in the frequencies and degrees of the TiO_2_ NPs-induced morphological changes was detected in the GEO+TiO_2_ NPs treated rats. The majority of acini appeared almost normal, with a significant decline in interstitial, inflammatory, and circulatory changes (Figure 7L). The prostatic lesion scoring among all groups is summarized in Table 3. 

## 4. Discussion 

Many consumer and commercial items are made using nanosized materials, which have unique physicochemical qualities such as high reactivity, color changes, lower melting temperatures, and better solar radiation absorption [40]. The widespread use of TiO_2_ NPs raises the risk of human exposure and environmental discharge, posing a potential health concern to humans, livestock, and the environment [41,42]. The toxicity of TiO_2_ NPs has been attributed primarily to the formation and accumulation of reactive oxygen species (ROS), which enhances an inflammatory response [7] and exhaustion of cellular antioxidants [8]. Geranium essential oil boasts a remarkable capacity to trap free radicals and restrict the production of new free radicals, thus lowering oxidative stress and inhibiting the development of several fatal consequences [17]. Therefore, the current study aimed to provide valuable data regarding Pelargonium graveolens essential oil components and their protective potential against TiO2 NPs-induced reprotoxicity in male Wistar rats.

The results of the GC-MS analysis of GEO revealed that citronellol (37.24%) and geraniol (12.32%) are the most prevalent chemicals found in GEO oil, followed by D-isomenthone, which is mainly reserved for fragrance, flavor, and pharma usage applications (6.17%), citronellyl ester (9.35%), and epi-γ-Eudesmol (6.87). There are numerous other components in addition to various fractions of several large components that are regarded as important antioxidants and flavonoids. These findings are consistent with previous studies [43], indicating that the GEO leaves contain high levels of these compounds.

In a living organism, oxidative stress is described as disrupting the balance between ROS formation and antioxidant defenses. Due to their unpaired electrons, ROS are highly reactive molecules created during normal cellular metabolism. Nevertheless, when produced in high concentrations, they can destroy cellular structures, lipids, proteins, and genetic material, contributing to a variety of pathological conditions. Several cellular antioxidant barriers, both enzymatic and non-enzymatic, exist to protect biological systems from free radicals. The tripeptide glutathione is one of the most critical defense mechanisms against ROS, particularly against H_2_O_2_ [44]. SODs are the first line of defense against harm caused by ROS. They catalyze the dismutation of superoxide anion free radical (O_2_^−^) into molecular oxygen and hydrogen peroxide (H_2_O_2_), thus reducing (O_2_^−^) levels, which are harmful to cells when present in high concentrations [45]. GPx and CAT are antioxidant enzymes that catalyze the conversion of H_2_O_2_ to water and oxygen. Oxidative stress is a significant factor for the development of male infertility, as the testicular tissue is highly predisposed to the activity of free radicals and oxidative stress due to several reasons, including high cell division rate, cell competition for oxygen rate, low oxygen pressure due to weakened vessels as well as high levels of unsaturated fatty acids [46]. 

Our results revealed that TiO_2_ NPs intraperitoneal administration induced testicular oxidant/antioxidant status alterations, as evidenced by a significant increase in testicular MDA level and a significant decrease in antioxidant enzymes (GPx, SOD, and CAT) activities as well as non-enzymatic antioxidant (GSH) content. TiO_2_ NPs cause excessive production of ROS in the testes of mice and rats. In contrast, the MDA level was promoted, and the levels of antioxidant-related enzymes SOD, GPx, and CAT were reduced, resulting in lipid peroxidation and cell apoptosis. This increased ROS in response to TiO_2_ NPs exposure also significantly impacts sperm because of the presence in the cell membrane of various polyunsaturated fatty acids [47]. According to growing data, ROS emission can disrupt the blood–testis barrier, worsening sperm dysfunction and impairing fertility [48]. Therefore, this could imply the high state of oxidative stress and altered state of semen picture after exposure to nanosized titanium in the current study. 

Moreover, the exposure of rats to TiO_2_ NPs for 60 days resulted in a significant reduction in the functional qualities of the sperm, as evidenced by a reduction in sperm count and motility, as well as a significant rise in morphological abnormalities. In addition, decreased number of germ cell types (spermatogonia, spermatocytes, and spermatids) and Sertoli cells were established. Different pathologies due to TiO_2_NPs treatment were manifested by degenerative alterations in the seminiferous tubules, epididymis, and prostate. Our results were supported by those of Morgan, Galal, Ogaly, Ibrahim, Abd-Elsalam and Noshy [5], who stated that TiO_2_ NPs administration induced a significant increase in testicular MDA levels and a significant decrease in GSH content and CAT activity as well as histological alterations of the testicular tissue, including interstitial edema and sloughing of its germinal epithelium with different apoptotic changes such as pyknosis, karyolysis, and karyorrhexis. Gao, et al. [48] reported Pathological changes involving sperm damage and degenerative and necrotic seminiferous tubules with apoptotic Sertoli cells in the testis of mice exposed to TiO_2_ NPs. 

In contrast, the supplementation of rats exposed to TiO_2_ NPs with the GEO induced a significant reduction in the frequencies and degrees of the TiO_2_ NPs-induced morphological changes in testicular, epididymal, and prostatic tissues and restored the antioxidant enzymes activity (SOD, GPx, CAT) as well as non-enzymatic antioxidant (GSH) content in the testicular tissue and reduced the level of MDA. This finding could be due to its immense components adopted in the presented data of the GC-MS profile of this oil. One of the best and most abundant components is geraniol, a potent antioxidant in many previous studies [49,50]. This impact might be attributed to its ability to prevent radicals from reaching their cellular components and targeting and quenching ROS by decomposing the free radicals, as well as the existence of monoterpenes, which have scavenging activity [51]. The presence of citronellal and citral in GEO may also contribute to the antioxidant properties of the plant. 

Evidence has accumulated that testicular steroidogenesis and spermatogenesis abnormalities are associated with male infertility caused by oxidative stress, as validated by a growing body of research. Sex steroid hormones share a common biosynthesis pathway with cholesterol and a unique precursor. STAR permits the cleavage of cholesterol into pregnenolone by mediating the transport of cholesterol from the outer mitochondrial membrane to the inner mitochondrial membrane [52]. CYP17A1 catalyzes when the conversion of pregnenolone is converted to dehydroepiandrosterone. Then, 17βHSD and 3βHSD enzymes ensure its conversion into androstenediol or androstenedione and testosterone [53,54]. The peroxisome proliferator-activated receptor-gamma coactivator 1-alpha (PGC-1α) performs a vital role in mitochondrial function and biogenesis control, including oxidative phosphorylation and reactive oxygen species detoxification [55]. In our data, the downregulation of all estimated expression of PGC-1α, 17ΒHSD, CYP17A1 and STAR genes was recorded in TiO2 NPs exposed group indicating the potential involvement of steroidogenesis (STAR, CYP17A1, and 17ΒHSD) and mitochondrial biogenesis (PGC)-related genes in the induction of testicular damage in toxicity with TiO_2_ NPs.

Leydig cells in the mammalian testis are responsible for testosterone production through stimulation by LH hormone, which is secreted by the pituitary gland in response to gonadotropin-releasing hormone (GnRH) from the hypothalamus [56]. Consequently, a reduction in testosterone level is a normal consequence of a reduction in LH level and oxidative damage of Leydig cells caused by ROS generation and depletion of antioxidant stores [57]. Therefore, the decreased levels of reproductive hormones, including TES, LH, and FSH, may be attributed to the ability of TiO_2_ NPs to pass the blood–testis barrier and accumulate in the testis, causing testicular lesions, sperm abnormalities, changes in serum sex hormone levels, alterations in the expression of genes related to spermatogenesis and hormone metabolic processes [49]. The previous authors recorded that TiO_2_ NPs administration induced a significant reduction in sperm numbers and motility, increased sperm abnormalities, and led to a substantial decline in serum TES, LH, and FSH in the TiO_2_ NPs-exposed group. Morgan, Galal, Ogaly, Ibrahim, Abd-Elsalam and Noshy [5] stated that TiO_2_ NPs administration induced a significant decrease in serum TES deteriorated spermiogram picture, elevated oxidative stress parameters, and upregulated the tested gene.

Notably, the current findings revealed that co-treatment of rats with GEO concurrently and TiO_2_ NPs resulted in a statistically significant elevation in TES and LH and increased the expression of the aforementioned genes. In addition, the GEO group had higher percentages of motile sperms and fewer sperm abnormalities close to the normal control level. This impact can be attributed to the increased capacity to quench free radicals due to the improved free radicals.

Patients diagnosed as infertile go through a great deal of emotional upheaval due to their condition. Infertile patients are at an increased risk of developing depression, anxiety, and discomfort [58]. According to some of the available research, oregano has been shown to significantly increase the motility and viability of human spermatozoa in laboratory studies. After 10 min of exposure, eucalyptus has also been shown to significantly increase the motility and viability of spermatozoa [59].

## 5. Conclusions

Our research demonstrated the potential involvement of oxidative stress as well as steroidogenesis (STAR, CYP17A1, and 17ΒHSD) and mitochondrial biogenesis (PGC)-related genes in the induction of testicular damage in toxicity with TiO_2_ NPs. Our findings suggested that administration of GEO could be beneficial in preventing nanosized titanium particles’ male reproductive disorders by increasing antioxidant capacity as well as regulating steroidogenesis and mitochondrial biogenesis-related genes, among other things. This study demonstrated that GEO could protect against testicular tissue damage caused by TiO_2_ NPs and the free radicals generated from these exposures. This finding may contribute to developing combinatorial treatments for infertility associated with various environmental and industrial xenobiotic exposures. 

However, there is a lack of availability to address some critical points in the current study regarding the mechanism by which GEO could exert its protective effect on the testicular tissue of rats. Consequently, further studies are required to overcome these limitations, improve fertility potential, and reduce the risk of toxicity with different environmental and industrial pollutants. 

## Figures and Tables

**Table 1:** Primer sequences, accession number, and product size for the quantitative RT-PCR for the analyzed genes in the testicular tissue.

Gene	Forward Primer (5′–3′)	Reverse Primer (5′–3′)	Accession No	Product Size
StAr	CCCAAATGTCAAGGAAATCA	AGGCATCTCCCCAAAGTG	NM_031558.3	187
CYP17A1	TGGCTTTCCTGGTGCACAATC	TGAAAGTTGGTGTTCGGCTGAAG	NM_012753.2	90
HSD17B3	AGTGTGTGAGGTTCTCCCGGTACCT	TACAACATTGAGTCCATGTCTGGCCAG	NM_054007.1	161
PGC1-α	ATGTGTCGCCTTCTTGCTCT	ATCTACTGCCTGGGGACCTT	NM_031347.1	180
GAPDH	GGCACAGTCAAGGCTGAGAATG	ATGGTGGTGAAGACGCCAGTA	NM_017008.4	143

StAr: steroidogenic acute regulatory protein; CYP17A1: cytochrome P450 Family 17 Subfamily A; HSD17B3: 17-beta hydroxysteroid dehydrogenase 3 Member 1; PGC-1α: peroxisome proliferator-activated receptor gamma coactivator 1-alpha; GAPDH: glyceraldehyde-3-phosphate dehydrogenase.

**Table 2:** GC–MS chromatogram of geranium oil, retention time (min) and peak area (%) of the various compounds assigned in the extract.

Peak	RT	Name	Formula	Area	Area Sum %
1.	24.299	Citronellol	C10H20O	55,776,528.03	37.24
2.	26.301	Geraniol	C10H18O	18,461,011.12	12.32
3.	19.967	Citronellyl ester	C11H20O2	14,007,012.3	9.35
4.	15.887	D-isomenthone	C10H18O	9,245,683.65	6.17
5.	3.476	Alpha.-Pinene	C10H16	1,391,254.48	0.93
6.	17.941	L-linalool	C10H18O	8,055,441.23	5.38
7.	11.807	CIS-ROSE OXIDE	C10H18O	1,926,217.6	1.29
8.	15.029	l-Menthone	C10H18O	3,170,974.01	2.12
9.	16.259	Alfa.-Copaene	C15H24	1,234,444.25	0.82
10.	17.014	Beta-Bourbonene	C15H24	2,648,338.41	1.77
11.	18.382	Isopulegol	C10H18O	699,337.32	0.47
12.	19.206	Caryophyllene	C15H24	2,651,769.58	1.77
13.	33.483	(epi-γ-Eudesmol)	C15H26O	10,293,442.99	6.87
14.	21.209	E,E-.alpha.-farnesene	C15H24	1,259,318.62	0.84
15.	21.461	Cis-Verbenol	C10H16O	937,277.21	0.63
16.	22.078	Gamma.-Elemene	C15H24	941,376.63	0.63
17.	22.262	Beta.-Myrcene	C10H16	4,132,866.08	2.76
18.	22.902	Citral	C10H16O	1,833,252.63	1.22
19.	23.795	Delta-Cadinene	C15H24	2,691,428.45	1.8
20.	25.546	Geranyl propionate	C13H22O2	630,509.85	0.42
21.	25.638	cis-Calamenene	C15H22	992,839.4	0.66
22.	27.12	α-Agarofurane	C15H24O	737,485.25	0.49
23.	27.52	Linalyl Acetate	C12H20O2	679,603.64	0.45
24.	31.846	Copaene	C15H24	892,608.23	0.6
25.	34.266	NERYL ACETATE	C12H20O2	1,582,583.89	1.06
26.	34.381	Spatulenol	C15H24O	838,394.74	0.56
27.	36.784	2-Phenylethyl tiglate	C13H16O2	1,228,021.02	0.82
28.	38.346	Aromandendrene	C15H24	848,453.07	0.57

**Table 3:** Lesion scoring in testicular, epididymal, and prostatic tissues of all groups.

Organ	Lesion	Control	GEO	TiO_2_	GEO + TiO_2_
Testes	Numbers of ST/10× microscopic field	14 ± 0.42 ^b^	13.7 ± 0.42 ^b^	18.5± 0.52 ^a^	17.3 ± 0.37 ^a^
	Mean diameters of ST (µm)	270.25 ± 7.23 ^a^	270.18 ± 6.3 ^a^	209.98 ± 5.1 ^c^	233 ± 9.4 ^b^
	Height of seminefrous (µm) (µm)epithelium	83.9 ± 0.85 ^a^	84.6 ± 0.9 ^a^	64 ±1.6 ^c^	74.7± 1.5 ^b^
	Numbers of permatogonia/ST	66.3 ± 4.9 ^a^	67.9 ± 4.4 ^a^	58.47 ± 3.06	63.8 ± 3.8
	Numbers of spermatocytes/ST	139.5 ± 1.7 ^a^	138.3 ± 2.4 ^a^ 1.7 ^a^	102.7 ± 3.7 ^c^	124.13 ± 3.4 ^b^
	Numbers of spermatids/ST	208.5 ± 2.9 ^a^	206.2 ± 4.8 ^a^	165.5 ± 8.1 ^c^	184.9 ± 8.1 ^b^
	Numbers of Sertoli/ST	17.3 ± 0.45 ^a^	17.6 ± 0.46 ^a^	15.3 ± 0.38 ^b^	16.6 ± 0.37 ^a^
	Numbers of ST with vacuolated epithelium	0 ± 0 ^c^	0 ± 0 ^c^	8.3 ± 0.29 ^a^	5.3 ± 0.56 ^b^
	Numbers of ST with necrotic epithelium	0 ± 0 ^c^	0 ± 0 ^c^	5.1 ± 0.53 ^a^	1.4 ± 0.2 ^b^
	Numbers of ST with depleted epithelium	0 ± 0 ^c^	0 ± 0 ^c^	5.4 ± 0.41 ^a^	1.8 ± 0.41 ^b^
	Numbers of ST with multinucleated giant cells	0 ± 0 ^c^	0 ± 0 ^c^	1.7 ± 0.16 ^a^	0.62 ± 0.21 ^b^
	Interstitial edema	0 ± 0 ^b^	0 ± 0 ^b^	6 ± 3 ^a^	2 ± 2 ^ab^
	Interstitial congestion	0 ± 0 ^b^	0 ± 0 ^b^	10± 3.3 ^a^	6 ± 3 ^ab^
Epididymis	Tubular irregularity	0 ± 0 ^b^	0 ± 0 ^b^	10 ± 3.3 ^a^	4 ± 2.6 ^ab^
	Absence of luminal sperms	0 ± 0	0 ± 0	6 ± 3	4 ± 2.6
	Presence of luminal round spermatids and/or exfoliated material	2 ± 2	2 ± 2	8 ± 3.2	4 ± 2.6
	Loss or disrupted of stereocilia	0 ± 0 ^b^	0 ± 0 ^b^	10 ± 3.3 ^a^	6 ± 3 ^ab^
	Vacuole formation	0 ± 0 ^b^	0 ± 0 ^b^	12 ± 3.2 ^a^	8 ± 3.2 ^a^
	Epithelial necrosis	0 ± 0	0 ± 0	6 ± 3.5	4 ± 2.6
	Hyperplastic alteration	0 ± 0	0 ± 0	0 ± 0	0 ± 0
	Interstitial inflammatory infiltrates	0 ± 0 ^b^	0 ± 0 ^b^	8 ± 3.2 ^a^	4 ± 2.6 ^ab^
	Interstitial vascular congestions	0 ± 0 ^b^	0 ± 0 ^b^	8 ± 3.2 ^a^	6 ± 3 ^ab^
	Interstitial hemorrhages	0 ± 0	0 ± 0	2 ± 2	0 ± 0
Prostate	Reduced or absence of luminal secretion	0 ± 0	0 ± 0	6 ± 3.05	4 ± 2.6
	Acinar dilatation	0 ± 0	0 ± 0	4 ± 2.6	4 ± 2.6
	Absence of the intra-acinar epithelial folds	2 ± 2 ^b^	2 ± 2 ^b^	12 ± 3.2 ^a^	6 ± 3.05 ^ab^
	Vacuolar degeneration	0 ± 0	0 ± 0	4 ± 2.6	2 ± 2
	Necrosis of the epithelial lining	0 ± 0 ^b^	0 ± 0 ^b^	8 ± 3.2 ^a^	4 ±2.6 ^ab^
	Hyperplasia of acinar epithelium	0 ± 0	0 ± 0	2 ± 2	0 ± 0
	Interstitial inflammatory cell infiltrate	0 ± 0	0 ± 0	6 ± 3.05	4 ± 2.6
	Increased connective tissue elements	0 ± 0	0 ± 0	6 ± 3.05	6 ± 3.05
	Interstitial congestion	0 ± 0 ^b^	0 ± 0 ^b^	10 ± 3.3 ^a^	6 ± 3 ^ab^
	Interstitial edema	0 ± 0	0 ± 0	6 ± 3.05	4 ± 2.6
	Interstitial hemorrhage	0 ± 0	0 ± 0	2 ± 2	2 ± 2

Data expressed as mean ± SE, N = 8 for each group. Each bar carrying different letters (a, b, and c) was significantly different at *p* < 0.05.
